# Supplementary figures and images for: 6-(Methylsulfonyl) Hexyl Isothiocyanate: A Chemopreventive Agent Inducing Autophagy in Leukemia Cell Lines
Source: Biomolecules. 2022 Oct 14;12(10):1485. doi: 10.3390/biom12101485 (PMC9599183; doi:10.3390/biom12101485)

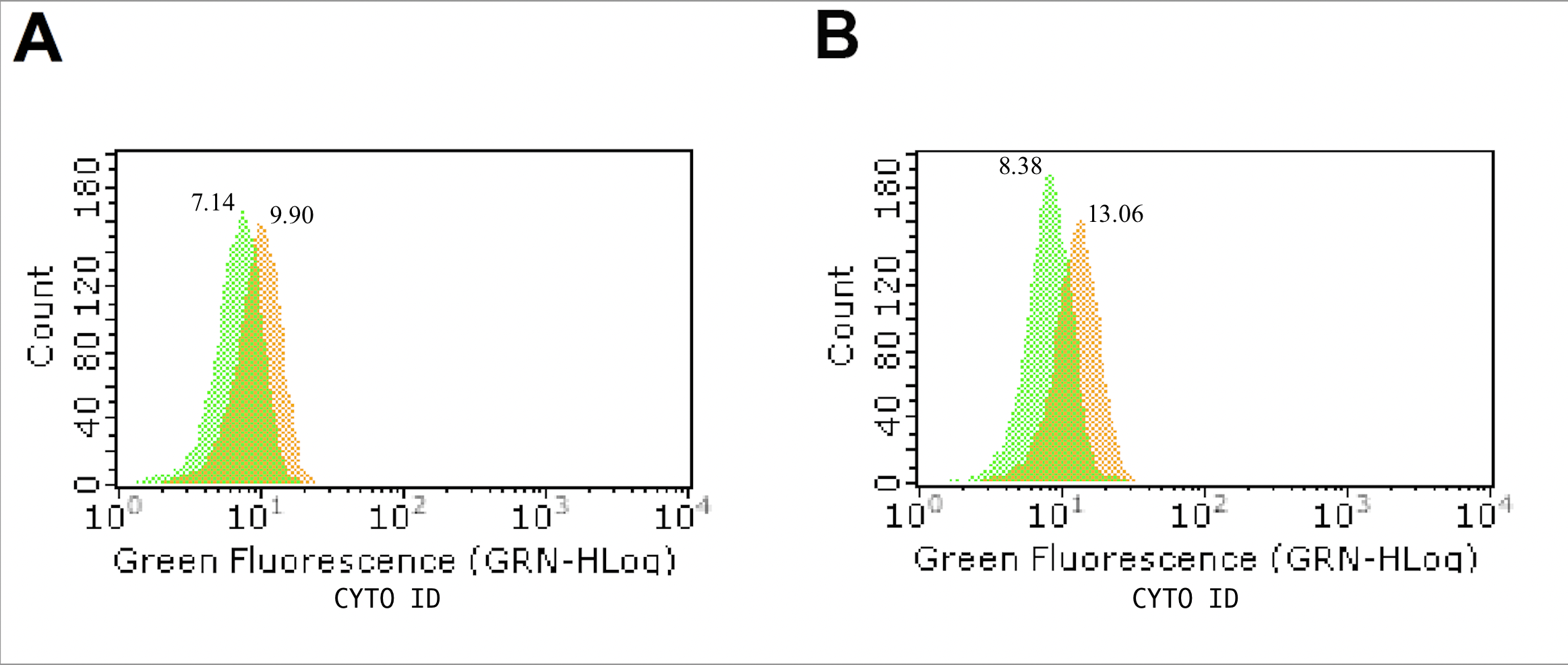

Supplement: Supplementary file 1 [file biomolecules-12-01485-s001.zip › biomolecules-1828926-Figure S1.png]
